# How does relative time in moderate-to-vigorous physical activity correspond to the 2020 guidelines on physical activity and sedentary behaviour?

**DOI:** 10.1186/s44167-023-00028-2

**Published:** 2023-10-02

**Authors:** Philip von Rosen

**Affiliations:** Department of Neurobiology, Care Sciences, and Society (NVS), Division of Physiotherapy, Karolinska Institutet, Huddinge, SE-141 83 Sweden

**Keywords:** Compositional data analysis, Guidelines, Moderate-to-vigorous physical activity, Physical activity, Recommendation

## Abstract

**Background:**

More and more researchers have started to analyse device-measured physical activity data using compositional data analysis (CoDA), which has led to that the effect of relative time in different behaviours can be explored. However, there are challenges related to the interpretation of the results based on CoDA. This is partly related to that CoDA provides estimates based on the relative time that is difficult to interpret relative to the 2020 guidelines of physical activity and sedentary behaviour. Since many data cohorts do not have data on sleep, the proportion of time in physical activity may vary depending on accelerometer wear time. Therefore, there is a need to explore cut-points for relative time to distinguish between individuals that do and do not reach 150–300 min of moderate-to-vigorous intensity physical activity (MVPA) per week. The aim was to establish a ratio of MVPA to awaken time that corresponds to meeting the 2020 guidelines of physical activity and sedentary behaviour in adults.

**Method:**

To estimate the cut-off points of relative time in MVPA, the publicly available data from NHANES 2003–2004 was used and cut-off points were explored in different subsets of the total population. Values for sensitivity, specificity and cut-off values were explored; i) in total sample, ii) by tertiles of wear time, iii) in individuals with ± 5 min from 150 to 300 min of MVPA, iiii) in individuals with ± 5 min from 150 to 300 min of MVPA in the middle tertile of wear time.

**Results:**

Overall, the analyses show high values for sensitivity (88–100%) and specificity (66–99%) for different cut-off values associated with 150–300 min of MVPA. Spending 2.4–4.7% of the time awake in MVPA was found to correspond to the 2020 guidelines of physical activity and sedentary behaviour.

**Conclusion:**

Based on publicly available data from NHANES 2003–2004, spending 2.4–4.7% of time awake in MVPA corresponds to meeting the 2020 guidelines of physical activity and sedentary behaviour.

## Background

The recent years, compositional data analysis (CoDA) has drawn significant attention from researchers in the field of physical activity, which is illustrated by a dramatical increase in the number of publications using CoDA for analysing device-measured physical activity. Combining the search terms “compositional data analysis” & “physical activity” in PubMed, shows a distinct increase in the number of publications; zero studies were published before 2015, one study was published in 2015 and between 2020 and 2022 37–41 studies were published each year. Clearly, researchers have started to apply CoDA to explore research aims related to device-measured physical activity data and different health outcomes. Since, device-measured physical activity can capture the full spectrum of physical activity intensity, it is important that we have methods to explore such data. CoDA could be used to explore the relative time of different combinations of physical activity intensity with different health outcomes.

Using CoDA, the co-dependent relationship among time in different behaviours such as sedentary behaviour, light-intensity physical activity and moderate-to-vigorous intensity physical activity (MVPA) is acknowledged [1]. Even if there are several other methodologies for handling the co-dependent relationship between different behaviour, i.e. related to isotemporal substitution [2], modified hierarchical regression or stratification by different behaviours [3], CoDA is one of the most flexible methods for this kind of data [4]. Even so, there are several challenges when using CoDA, where one of these is related to the interpretation of the results.

It is well known that engaging in physical activity is associated with health benefits (e.g. cognitive health, sleep, etc) and does mitigate health risks such as the reduced risk of all-cause mortality, cardiovascular disease mortality, incident hypertension, cancer, etc [5, 6]. The 2020 guidelines of physical activity and sedentary behaviour (now called “2020 guidelines of PA and SB”) emphasize that adults should undertake 150–300 min of moderate-intensity physical activity, or 75–150 min of vigorous-intensity physical activity, or some equivalent combination of moderate-intensity and vigorous-intensity aerobic physical activity, per week [7]. The guideline also emphasizes that ”some physical activity is better than none” and “adults should limit the amount of time spent being sedentary” are the main messages.

The 2020 guidelines of PA and SB refer to absolute time while disregarding a recommendation based on relative time. There are also 24-hour guidelines based on physical activity, sedentary behaviour and sleep, such as the Canadian 24-Hour Movement Guidelines for Adults [8] and the Australian 24-Hour Movement Guidelines for Children and Young People [9]. Although these provide 24-hour guidelines, these recommendations do not provide guidelines based on relative values. As the relative time is affected by the time in all behaviours it is more difficult to provide an optimal composition about how an individual should spend their day from a health perspective. In addition, there are far more publications on absolute time of physical activity, compared to relative time [10]. Applying CoDA, leads normally to an output of relative time for a specific behaviour, which is often quite difficult to interpret relative to the 2020 guidelines of PA and SB. Even if percent could be transformed to absolute time, and rescaled to sum up to 24 h [11], identifying reference values associated with the 2020 guidelines of PA and SB may help in interpreting which values correspond to the strongest health benefits. It might also help when visualizing diagrams, e.g. ternary plots [12], where relative values are depicted.

If data on complete 24 h is available, the recommended guidelines of 150–300 min should correspond to that an adult individual should spend between 1.5 and 3.0% of total time in MVPA. However, in most cases, data on sleep is not recorded and only time in awaken behaviours can be modelled. If not 24 h of data is recorded, the fraction of MVPA may vary depending on how large part of the day in awaken behaviours is recorded. Considering that most guidelines recommend including days with at least 10 h of valid data [13], it is important to estimate how this could influence the relative time of MVPA that corresponds to the 2020 guidelines of PA and SB. For instance, if sleep time is ignored and an individual spend 30 min in MVPA, the proportion of time will vary between 3.1 and 6.3% if awaken time is between 8 and 16 h. Since participants in a study often have collected data across different wear time it is important to estimate which cut-off values can identify most individuals reaching the recommendation (sensitivity) and distinguish between the ones that do not achieve the recommendation (specificity). Therefore, the aim was to establish a ratio of MVPA to awaken time that corresponds to meeting the 2020 guidelines of PA and SB in adults.

## Method

To explore the aim of this study, the publicly available data of NHANES 2003–2004 was used. Details on data collection of NHANES 2003–2004 have previously been described [14].

In short, participants wore an ActiGraph 7164 accelerometer (ActiGraph, Shalimar, FL) on the right hip for seven consecutive days, to capture time in different intensities. The device was set to sampling counts per 1-minute epochs and non-wear time was defined as periods of at least 60 consecutive min of zero counts. Accelerometer data were treated and extracted using the nhanesaccel package for R *(*release 4.1.3; R Core Team, 2015, Vienna, Austria*).* The nhanesaccel package generates measures of activity volume, intensity and frequency according to specified criteria. In this analysis, only time spent in moderate to vigorous physical activity (≥ 760 counts) was extracted, since an equivalent combination of moderate-intensity and vigorous-intensity aerobic physical activity can be used to meet the 2020 guidelines of PA and SB. A valid day was defined as 10 or more hours of wearing an accelerometer and participants with records of 4 or more valid days were included. This resulted in a sample of 4154 participants.

To establish cut-off points associated with meeting the 2020 guidelines of PA and SB, the Youden score [15], was used to find an optimal trade-off between sensitivity and specificity. Several different subsets of the total population were used when estimating cut-off values that correspond to 150–300 min of MVPA. Specifically, cut-off values, defined as proportion time spent in MVPA (in percent), were explored; (i) in the total sample, (ii) by tertiles of wear time, (iii) in individuals with ± 5 min from 150 to 300 min of MVPA, iiii) in individuals with ± 5 min from 150 to 300 min of MVPA in the middle tertile of wear time. This was done to investigate if the cut-off values varied across wear time and for the sample that met the 2020 guidelines of PA and SB. Accounting for the complex survey design of NHANES, values for sensitivity and specificity were explored in the package WeightedROC in the statistical software R.

To provide an example when the proportion time spent in MVPA is not constant across wear time, a random sample of data was generated in the statistical software R. Using “set.seed (1234)” with function “rnorm” to create reproducible results, data on wear time and proportion time spent in MVPA were generated. For wear time, the mean was set to “700”, “850” and “1050” min with a corresponding standard deviation of “50”, “100” and “100” min, respectively. For the proportion time spent in MVPA, the mean was set to “0.03”, “0.03” and “0.04”, with a corresponding standard deviation of “0.01”, “0.015” and “0.02”, respectively.

## Results

Overall, the analyses showed very high values for sensitivity and specificity for different cut-off values associated with the 2020 guidelines of PA and SB (Table 1). The lowest sensitivity (88%) and specificity (66–85%) values were associated with individuals that were ± 5 min from the 2020 guidelines of PA and SB. Taking the median value of the cut-off values showed that meeting the 2020 guidelines of PA and SB corresponds to that 2.4–4.7% of awaken time is spent in MVPA.

**Table 1:** Sensitivity, specificity, cut-off values for reaching 150–300 min of moderate-to-vigorous intensity physical activity (MVPA) per week for different subset of sample based on wear-time and minutes of MVPA.

	150 min of MVPA^a^	Sensitivity (%)	Specificity (%)	Cut-off MPA (%)	300 min of MVPA^a^	Sensitivity (%)	Specificity (%)	Cut-off MVPA (%)
Complete sample	1128	98	98	2.4	387	99	97	4.4
**Wear-time**								
<33th percentile	245	99	98	2.6	74	100	99	5.5
33 to 67th percentile	383	99	99	2.5	139	100	99	4.7
≥67th percentile	500	98	99	2.3	174	98	98	4.4
± 5 min from 150–300 min of MVPA^b^	251	88	85	2.4	90	88	66	4.7
**Wear-time**								
≥33 to 67th percentile and ± 5 min from 150–300 min of MVPA^b^	82	95	97	2.5	38	96	87	4.8

^a^ Reaching the recommended level of physical activity (150–300 min of MVPA per week).

^b^ 145–305 min of MVPA per week.

**Fig. 1:**
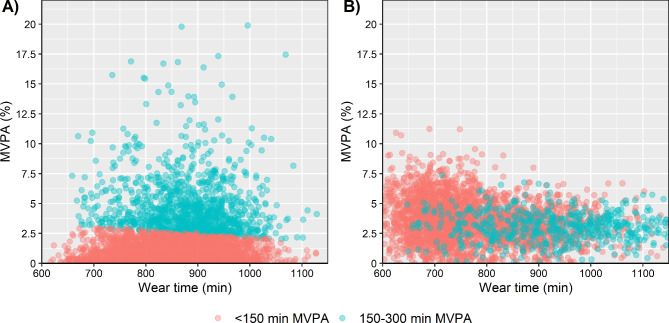
Relative time (%) spent in moderate-to-vigorous intensity physical activity (MVPA) per week across wear time by reaching the recommended level of 150–300 min of MVPA per week, for **(A)** data from NHANES 2003–2004 and **(B)** randomly generated data

If the relative time of a behaviour deviates across wear time it could be suspected that the time in a behaviour is biased. In Fig. 1A, based on publicly available data from NHANES 2003–2004, the proportion of time spent in MVPA and reaching the recommended level of the 2020 guidelines of PA and SB across wear time were rather constant. In Fig. 1B, based on randomly generated data, the individuals that reached the recommended level of the 2020 guidelines of PA and SB had the lowest amount of wear time, illustrating that time in at least one behaviour is not accurately estimated. The figure also highlights that the proportion of individuals reaching the recommended level of the 2020 guidelines of PA and SB is constant across wear time when time in different behaviours is unbiased.

## Discussion

The 2020 guidelines of PA and SB for adults specify now a target range of MVPA [7]. The cut-off findings should be interpreted that an individual should spend between 2.4 and 4.7% of awaken time in MVPA to reach this target range. There was no dramatic change in cut-off values across different values for wear time, indicating that the derived cut-off values are quite robust. If time in different behaviours is unbiased, estimates of relative time will be correct as well. However, if time is classified as non-wear time that corresponds to time spent in a behaviour, the estimates of relative time will be inaccurate. It is important to point out that the proposed cut-off interval may not be accurate in other populations than adults. Even so, the cut-off interval overlaps with the 1.5-3.0% range of MVPA that is associated with meeting the 2020 guidelines of PA and SB in individuals having 24 h of data per day available. This suggests that a wider range of relative time spent in MVPA should be allowed when investigating data on only awaken behaviours.

## Conclusion

In summary, more and more researchers have started to analyse device-measured physical activity data using CoDA. However, there are challenges related to the interpretation of the results, partly related to that CoDA provides estimates based on relative time, which is not applicable to the 2020 guidelines of PA and SB. Based on estimations of cut-points using different subsets of the NHANES 2003–2004 population, spending 2.4–4.7% of the time awake in MVPA was found to correspond to 150–300 min of physical activity. This finding could help when analysing physical activity data using CODA in datasets where sleep is not available and when interpreting diagrams depicting relative time in different behaviours.

## Data Availability

Data are available in a public, open-access repository and can be accessed at the https://www.cdc.gov/nchs/nhanes/index.htm.
